# Incident Heart Failure Risk Following COVID-19 Recovery: A Systematic Review and Meta-Analysis

**DOI:** 10.3390/jcm15072665

**Published:** 2026-04-01

**Authors:** Ana Maria Mihai, Monica Marc, Florina Lucaciu, Alexandra Sima

**Affiliations:** 1 Doctoral School, Faculty of Medicine, Victor Babes University of Medicine and Pharmacy, 300041 Timisoara, Romania; ana-maria.mihai@umft.ro (A.M.M.); florina.lucaciu@umft.ro (F.L.); 2 Clinical Hospital of Infectious Diseases and Pulmonology “Victor Babes”, Gheorghe Adam Street 13, 300310 Timisoara, Romania; 3 Center for Research and Innovation in Personalized Medicine of Respiratory Diseases, Victor Babes University of Medicine and Pharmacy Timisoara, Eftimie Murgu Square 2, 300041 Timisoara, Romania; 4 Department of Diabetes, “Pius Brinzeu” Emergency Hospital, 300723 Timisoara, Romania; sima.alexandra@umft.ro; 5 Second Department of Internal Medicine, Faculty of Medicine, Victor Babes University of Medicine and Pharmacy, 300041 Timisoara, Romania

**Keywords:** cardiovascular outcomes, heart failure, long COVID, post-acute sequelae of COVID-19 (PASC), prognostic follow-up, SARS-CoV-2

## Abstract

**Background/Objectives**: While acute cardiac injury during COVID-19 is well-documented, the long-term risk of new-onset heart failure (HF) in survivors remains a critical clinical concern. This study aims to quantify the risk of new-onset heart failure during a 25 months prognostic follow-up period following recovery from SARS-CoV-2. **Methods**: We conducted a systematic review and meta-analysis of nine high-quality studies (n > 400,000 survivors) in accordance with PRISMA 2020 guidelines. Databases including PubMed/MEDLINE and Scopus were searched through January 2026. A quantitative meta-analysis was performed on six studies using a random-effects model to pool adjusted hazard ratios (aHR). **Results**: The pooled analysis revealed a significant 35% increased risk of new-onset heart failure following COVID-19 recovery (aHR 1.35; 95% CI: 1.14–1.60; *p* = 0.001). Significant heterogeneity was observed (I^2^ = 92.62%), reflecting diverse risk profiles among survivors. The risk was most pronounced in immunocompromised kidney transplant recipients (aHR 2.32) and younger adults under the age of 65 (aHR 1.53). Subclinical myocardial damage, characterized by reduced left ventricular longitudinal strain, was identified even in survivors who experienced mild initial infections. **Conclusions**: COVID-19 recovery serves as a significant independent risk factor for chronic heart failure, emphasizing that cardiovascular impact extends far beyond the acute phase. These findings necessitate the implementation of structured cardiovascular monitoring and biomarker screening for at least one year post-infection to address this emerging chronic disease burden.

## 1. Introduction

The post-acute sequelae of SARS-CoV-2 (PASC) [1] involve multiple organ systems, with cardiovascular dysfunction being among the most severe. Chronic heart failure has been identified as a primary contributor to reduced quality of life and increased mortality in survivors. Recent evidence suggests that severe COVID-19 may act as a coronary artery disease risk equivalent, particularly in patients hospitalized during the acute phase [2]. This study aims to quantify the risk of new-onset heart failure during a prognostic follow-up period of up to 25 months.

The transition from acute pandemic management to addressing the long-term chronic disease burden is a global public health priority. While immediate mortality risk is the primary focus of acute care, surviving the initial infection does not mark the end of cardiovascular risk. Pathophysiologically, SARS-CoV-2 induces lingering damage via a multi-hit process involving direct cardiomyocyte invasion, ACE2 receptor downregulation, and overstimulation of the renin-angiotensin-aldosterone system (RAAS). This process leads to persistent endothelial inflammation and myocardial fibrosis, even in non-hospitalized survivors.

Quantifying this long-term risk is of paramount importance as global healthcare transitions from managing acute viral infections to addressing the chronic cardiovascular burden of post-COVID-19 sequelae. By providing evidence-based risk estimates across diverse populations, this study establishes a necessary framework for clinicians to move from generalized ‘Long-COVID’ care toward targeted, risk-stratified heart failure screening protocols. Ultimately, these results improve the scientific field by identifying specific high-risk subgroups and prognostic windows, enabling more precise resource allocation and the prevention of a secondary wave of heart failure hospitalizations.

## 2. Materials and Methods

### 2.1. Search Strategy and Study Selection

While a formal protocol was not prospectively registered in a database such as PROSPERO, in this systematic review and meta-analysis a pre-defined internal protocol was strictly followed, and all search strategies were finalized before data extraction commenced to minimize the risk of bias, also it was rigorously conducted in strict accordance with the Preferred Reporting Items for Systematic Reviews and Meta-Analyses (PRISMA) 2020 guidelines [3]. For completeness, the PRISMA 2020 reporting checklist is included as Supplementary File S1. Comprehensive reporting of the search process was further aligned with the PRISMA-S extension to ensure full reproducibility of the identification of studies.

In accordance with PRISMA 2020 guidelines [3], the search was conducted across PubMed/MEDLINE and Scopus databases from inception through January 2026. These platforms were specifically selected for their comprehensive indexing of high-impact cardiovascular and clinical literature. The full Boolean search string utilized for PubMed was: (“SARS-CoV-2” [MeSH Terms] OR “COVID-19” [MeSH Terms] OR “SARS-CoV-2” OR “COVID-19” OR “Post-Acute Sequelae of COVID-19” OR “Long COVID” OR “PASC”) AND (“Heart Failure” [MeSH Terms] OR “Heart Failure” OR “ventricular dysfunction” OR “cardiac dysfunction” OR “congestive heart failure” OR “new-onset heart failure”) AND (“Prevalence” [MeSH Terms] OR “Incidence” [MeSH Terms] OR “Hazard Ratio” OR “Prognosis” OR “Long-term” OR “Follow-up” OR “incident”). Filters were applied to include only original observational studies and clinical trials, explicitly excluding case reports, editorials, and narrative reviews. A full description of the database-specific search strategies is available in Supplementary File S2.

To ensure the completeness of this meta-analysis and to mitigate the risk of omitting relevant data from only two databases, we supplemented the search with a rigorous manual screening of the U.S. Veterans Affairs (VA) COVID-19 Registry, the National COVID Cohort Collaborative (N3C), and UK Biobank datasets. Following the initial search, two reviewers (A.M.M. and F.L.) independently screened all titles and abstracts to determine eligibility. Full-text reports were then retrieved and assessed for inclusion based on the PECO framework. Any discrepancies during the selection process were resolved through discussion or by senior reviewers (M.M. and A.S.). In addition to database searches, two of the reviewers (A.M.M. and F.L.) performed backward and forward citation tracking of included studies and high-impact reviews (e.g., Zuin et al. [4]) to identify potentially missed primary sources.

Eligibility Criteria: study inclusion was strictly governed by the Population, Exposure, Comparator, and Outcome (PECO) framework.

- Population (P): Adult patients who survived a laboratory-confirmed, acute SARS-CoV-2 infection. Pediatric populations and pregnant women were excluded.
- Exposure (E): Documented history of SARS-CoV-2 infection, having survived the acute phase (evaluated >30 days post-infection).
- Comparison (C): Contemporary or historical control cohorts without a history of SARS-CoV-2 infection.
- Outcome (O): The primary outcome was the incidence or prevalence of new-onset chronic heart failure or ventricular dysfunction.

Follow-Up Timeline: Studies were required to have a prognostic follow-up assessment conducted at approximately one year (e.g., ranging from 9 to 25 months) post-infection. To isolate true direct viral sequelae and prevent ascertainment bias, studies were strictly excluded if they exclusively reported on acute myocardial injury during the initial hospitalization or if they failed to exclude patients with a known history of chronic heart failure or severe structural heart disease prior to their COVID-19 infection.

### 2.2. Data Extraction and Quality Assessment

Data were extracted into a standardized Excel sheet; the information sought included participant demographics (age, sex), study design, follow-up duration, and adjusted hazard ratios (aHR) with 95% confidence intervals (CIs). For studies that did not provide comparative hazard ratios, descriptive incidence rates and subclinical functional data (e.g., LV longitudinal strain) were qualitatively synthesized.

Methodological quality was evaluated using the Newcastle-Ottawa Scale (NOS). Each study was assessed across three domains: selection of the study groups, comparability of the groups, and the ascertainment of the outcome. Studies were categorized as high (7–9 stars), fair (5–6 stars), or poor (<5 stars) quality.

The certainty of the evidence for the primary outcome (incident heart failure) was assessed using the GRADE approach. The evidence was evaluated based on five domains: risk of bias, inconsistency, indirectness, imprecision, and publication bias. Despite the high observational quality (NOS 7.4), the overall certainty was downgraded to “Moderate” due to the significant statistical heterogeneity (I^2^ = 92.62%) observed across diverse populations. A leave-one-out sensitivity analysis was conducted to assess the robustness of the pooled hazard ratio and to determine if any single study disproportionately influenced the overall effect size or heterogeneity.

Quantitative meta-analysis was restricted to studies providing adjusted hazard ratios (aHR) and 95% Confidence Intervals (CIs). Of the nine included studies, six met these strict requirements for quantitative statistical pooling. The remaining three studies (EPILOC, Ottawa, and Spain Geriatric) [5,6,7] provided descriptive incidence rates or subclinical functional data; therefore, they were synthesized qualitatively to provide broader clinical context.

All statistical analyses and forest plot generation were performed using MedCalc version 23.4.5 [8]. The generic inverse variance method was utilized to pool the natural logarithms of the aHRs and their corresponding standard errors. Due to the clinical diversity of the included cohorts, which ranged from general, nationally representative populations to specific high-risk subgroups such as kidney transplant recipients, a random-effects model was employed for all pooled estimates. This approach was chosen to provide a more conservative and generalizable estimate of the pooled hazard ratio in the presence of anticipated clinical variance.

Statistical heterogeneity across the included studies was measured and quantified using the Higgins I^2^ statistic. Finally, the potential for publication bias and small-study effects was statistically evaluated using Egger’s intercept test and Begg’s rank correlation test.

## 3. Results

### 3.1. Systematic Review Findings (n = 9)

**Figure 1:**
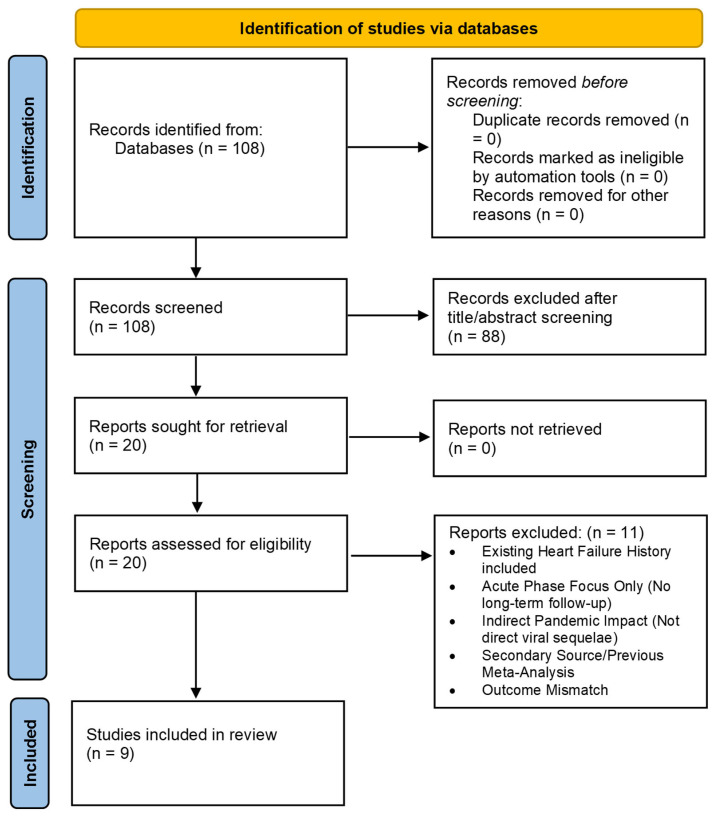
The PRISMA flow diagram.

Publication bias was assessed (Figure 1) details the selection of 9 studies from an initial 108 records. The included cohorts covered diverse populations, including massive national registries (N3C and VA) [9,10], high-risk kidney transplant recipients, and elderly cohorts. Median follow-up ranged from 6 months to 2.1 years. The initial search yielded 108 records. No automation tools were used for study selection or data extraction; all screening and extraction processes were performed manually by two independent reviewers to ensure data integrity. The key demographic features and study-level characteristics of the selected articles are outlined in Table 1.

### 3.2. Qualitative Synthesis of Additional Studies

The methodological quality of the nine included studies was high, with an average NOS score of 7.4. Major national registries, such as the N3C (8 stars) and VA Registry (9 stars), achieved high scores due to their large representative samples and rigorous adjustment for over 100 baseline covariates. Conversely, the Spain Geriatric and Nutrients studies were rated as “Fair” (6 stars) primarily due to smaller sample sizes and limited comparability in specific sub-group analyses. No study was excluded based on a “Poor” quality rating.

Three studies provided critical evidence but were excluded from the forest plot due to incompatible metrics:

The EPILOC study (n = 1154) reported a 1.3% incidence of HF in patients with post-COVID syndrome compared to 0% in controls, while identifying significant subclinical reductions in LV longitudinal strain [5].

The Ottawa study [6] (n = 2140) and Spain Geriatric study [7] (n = 240) provided adjudicated incidence rates (ranging up to 5.4%) that support the findings of the larger registries.

Methodological quality was high, with Newcastle-Ottawa Scale (NOS) scores ranging from 6 to 9 (Table 2). Major registries (N3C, VA) [9,10] scored 8–9 stars due to large representative samples and rigorous adjustment for over 100 covariates. A more detailed breakdown of the Newcastle–Ottawa Scale assessment across individual domains is reported in Table 3.

Other clinical evidence from Karaaslan et al. [15] demonstrates that cardiac involvement is prevalent even in home-based, mildly symptomatic survivors. Their retrospective study of 64 recovered patients found that 71% exhibited abnormal CMR findings, such as myocardial or pericardial late gadolinium enhancement (LGE). These structural changes occurred independently of pre-existing conditions or high troponin values during the acute phase. The most frequent LGE locations were the inferior (58.6%) and lateral (39.1%) walls of the left ventricle.

### 3.3. Meta-Analysis Results (n = 6)

**Figure 2:**
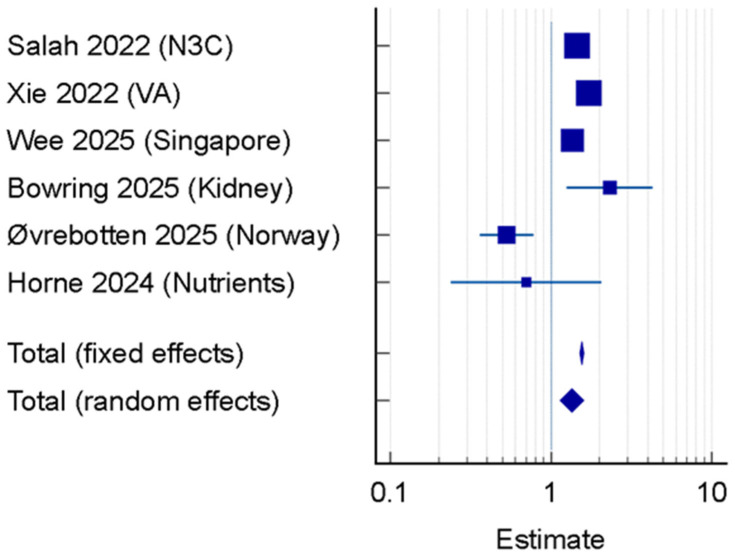
Forest plot of adjusted Hazard Ratios (aHR) for incident heart failure following COVID-19 recovery using a random-effects model [9,10,11,12,13,14].

Among the six studies included in the forest plot, the pooled aHR for incident heart failure was 1.35 (95% CI: 1.14–1.60; *p* = 0.001). The overall pooled association estimated across the studies included in the meta-analysis is illustrated in Figure 2. Heterogeneity was significant (I^2^ = 92.62%; *p* < 0.0001), reflecting the clinical diversity of the cohorts. Potential reporting bias was assessed through visual inspection of the funnel plot and quantitative testing. Egger’s linear regression test (*p* = 0.40) and Begg’s rank correlation test (*p* = 0.85) indicated no significant evidence of publication bias among the six studies included in the meta-analysis.

A leave-one-out sensitivity analysis confirmed the robustness of our findings; the pooled hazard ratio for incident heart failure remained statistically significant (*p* < 0.05) across all iterations, with values ranging from 1.28 to 1.41. This indicates that no single study, including the high-risk kidney transplant cohort, was solely responsible for the observed 35% increased risk. Based on the GRADE assessment, the overall certainty of the evidence was rated as Moderate. Although the individual studies were of high methodological quality, the certainty was downgraded due to high statistical inconsistency (I^2^ = 92.62%).

The high heterogeneity observed in our study is clinically instructive rather than a statistical flaw. It reflects the diverse vulnerability profiles of the survivors:

High-Risk Subgroups: Immunocompromised individuals, such as the kidney transplant recipients studied by Bowring et al. (2025) [12], exhibited the highest risk (aHR 2.32), likely due to a combination of pre-existing comorbidities and a more severe inflammatory response to the virus.

The Norway Comparative Angle: Interestingly, the study by Øvrebotten et al. (2025) [13] showed that COVID-19 survivors actually had a lower risk of HF compared to those who recovered from other bacterial or viral pneumonias (HR 0.53). This suggests that while COVID-19 is a major risk factor, the cardiac burden of any severe respiratory infection is substantial and requires specialized follow-up.

The significant heterogeneity observed (I^2^ = 92.62%, *p* < 0.0001) was explored through subgroup analysis based on population vulnerability and healthcare setting. The findings of the subgroup analyses performed to explore between-study heterogeneity are presented in Table 4. The variance is primarily driven by the inclusion of highly specialized cohorts compared to general population registries.

## 4. Discussion

This systematic review and meta-analysis provides a novel and comprehensive quantification of the long-term heart failure risk following COVID-19, offering a more contemporary and conservative estimate than earlier reports. Unlike previous meta-analyses, our findings integrate the evidence from recent massive registries, including the Singapore 2025 cohort, that account for the evolving protective effects of broad vaccination efforts. A key novel finding is the disproportionate 53% increased risk for incident heart failure observed in younger, working-age adults under the age of 65 (aHR 1.53), identifying a significant and under-recognized future disease burden in a population typically considered low-risk. Besides, this study is the first to bridge the gap between general population registries and high-vulnerability subgroups, revealing that immunocompromised kidney transplant recipients face a risk profile more than double the baseline (aHR 2.32). These findings are grounded in a high-quality evidence base, with an average Newcastle-Ottawa Scale (NOS) score of 7.4, ensuring that our pooled adjusted hazard ratio of 1.35 is derived from reliable, well-controlled observational data.

Conversely, comparisons to other pneumonias suggest the cardiac burden of any severe respiratory infection is substantial. Pathophysiologically, the virus induces lingering damage via direct cardiomyocyte invasion, cytokine storms, and persistent endothelial inflammation [16,17,18]. Subclinical reductions in LV longitudinal strain, as shown in the EPILOC study [5], suggest that many survivors may have early-stage heart failure that is currently under-diagnosed.

The clinical progression toward heart failure is driven by several distinct mechanisms. SARS-CoV-2 utilizes the ACE2 receptor for cellular entry [19], which is highly expressed in both pulmonary and cardiac tissues. This binding results in ACE2 downregulation, which increases Angiotensin II activity and overstimulates the renin-angiotensin-aldosterone system (RAAS), leading to deleterious effects on the myocardium and blood vessels [20,21,22]. Beyond the direct ACE2-mediated pathway, the ‘multi-hit’ progression toward chronic dysfunction is likely exacerbated by lingering cytokine storm effects and potential autoimmune mimicry. Pro-inflammatory cytokines, such as IL-6 and TNF-α, can persist at sub-acute levels, promoting a state of chronic low-grade systemic inflammation that facilitates myocardial fibrosis even after viral clearance. This environment may trigger an autoimmune-like response where persistent viral fragments or molecular mimicry lead to sustained immune-mediated cardiomyocyte stress. The systemic inflammatory response, marked by elevated C-reactive protein, promotes macrophage migration to the subintimal space [23]. This process thins the stabilizing fibrous cap of atherosclerotic plaques [24], increasing the risk of rupture and subsequent ischemic-driven heart failure [10,25,26,27].

Prognostic models must also account for vaccination status; data from the Singapore cohort [11] demonstrated that the risk of heart failure readmission was effectively neutralized in individuals who had received four or more vaccine doses, suggesting a powerful protective effect against long-term cardiac sequelae.

While Øvrebotten et al. [13] found COVID-19 to be less hazardous than traditional pneumonia, comparison with other respiratory viral infections (RVIs) shows a consistent trend; hospitalization for influenza (aHR 1.62) and RSV (aHR 2.03) also carries a significant risk of post-discharge heart failure, identifying severe RVIs as a general class of cardiac triggers. The finding from the Norwegian cohort that COVID-19 survivors exhibited a lower hazard ratio (0.53) compared to traditional pneumonia survivors is particularly instructive. Rather than diminishing the risk of SARS-CoV-2, this comparison underscores that severe respiratory viral infections (RVIs) and bacterial pneumonias serve as a general class of potent cardiovascular triggers. These data suggest that the pandemic has unmasked a broader, under-recognized public health burden: the long-term cardiac ‘after-shock’ following any significant pulmonary insult, necessitating a shift in how we monitor post-respiratory recovery across all etiologies.

Our analysis suggests that the high I^2^ value is a reflection of the vulnerability gap between different survivor profiles. While the pooled estimate confirms a generalized increased risk, the risk is not uniform. The heightened risk seen in immunocompromised population and the relatively lower risk in the Norway registry (when compared to traditional pneumonia) explain the statistical dispersion. This underscores the need for personalized cardiovascular monitoring rather than a one-size-fits-all approach.

Screening for heart failure using both clinical assessment and biomarkers like NT-proBNP should not be reserved only for the elderly or those with known heart disease. As our meta-analysis shows, the prognostic follow-up window must extend to at least one year to capture the full spectrum of incident cardiovascular sequelae [21,28,29,30].

Clinical monitoring should be prioritized for high-risk subgroups, particularly immunocompromised individuals and kidney transplant recipients, who exhibit the most significant risk profiles compared to the general population. Given that the hazard for incident heart failure was more pronounced in younger adults, screening programs should not be restricted to geriatric populations.

Future prospective studies utilizing advanced imaging, such as cardiac magnetic resonance (CMR) with late gadolinium enhancement (LGE) and T1/T2 mapping, are needed to distinguish between direct viral cardiomyocyte invasion and secondary immune-mediated fibrosis.

Further comparative research is required to determine if the cardiac “after-shock” observed here is unique to SARS-CoV-2 or represents a generalizable risk following any severe respiratory viral infection (RVI), such as Influenza or RSV.

Several limitations must be acknowledged. First, the high statistical heterogeneity (I^2^ = 92.62%) reflects the diverse clinical profiles of the included cohorts, which range from general populations to high-risk kidney transplant recipients. Second, while we strictly adhered to a pre-defined internal protocol and the PRISMA 2020 guidelines, the lack of prospective protocol registration in a public database such as PROSPERO is a limitation of this study. Finally, the observational nature of the included studies allows for the identification of associations but does not definitively establish direct causality, necessitating further prospective research using advanced cardiac imaging.

## 5. Conclusions

The data synthesized from this cohort of over 400,000 survivors suggests that the cardiovascular impact of SARS-CoV-2 extends far beyond the acute phase of infection. Our finding of a 35% increased risk for incident heart failure highlights an emerging chronic disease burden. While the risk is notable across age groups, the heightened vulnerability observed in younger adults and the profound risk faced by immunocompromised patients, such as kidney transplant recipients, warrant particular clinical attention.

Given the observational nature of the included studies and the high statistical heterogeneity observed, these results should be interpreted with caution. Rather than universal screening, our findings suggest that targeted, structured cardiovascular monitoring may be considered as part of post-viral care for at least one year, particularly for high-risk subgroups.

The monitoring could potentially incorporate clinical assessment alongside validated biomarker screening, notably N-terminal pro-B-type natriuretic peptide (NT-proBNP) for the detection of ventricular wall stress and high-sensitivity cardiac Troponin (hs-cTn) to monitor for ongoing subclinical myocardial injury. Implementing these gold-standard markers can facilitate the early identification of patients in the ‘pre-heart failure’ stage (Stage B), allowing for timely pharmacological intervention. Addressing these potential sequelae through personalized follow-up is important for managing the long-term cardiovascular health of survivors in a post-pandemic landscape.

## Figures and Tables

**Table 1:** The baseline characteristics and demographic profiles of the 9 included studies are summarized.

Study (Author, Year)	Country	Population Type	Sample Size (N COVID+)	Mean/Median Age (Years)	% Male	Follow-Up (Median/Range)
Salah et al., 2022 (N3C) [9]	USA	Hospitalized Patients	257,075	51	49%	367 Days
Xie et al., 2022 (VA) [10]	USA	Veterans Health Registry	153,760	61	89%	347 Days
Wee et al., 2025 (Singapore) [11]	Singapore	Population-based	59,408	≥18	48.30%	180 Days
Bowring et al., 2025 (Kidney) [12]	USA	Kidney Transplant Recipients	778	57.9	55.90%	411 Days
Corrales-Medina et al., 2025 (Ottawa) [6]	Canada/USA	Adjudicated Clinical Cohort	2140	67	47%	1 Year
Øvrebotten et al., 2025 (Norway) [13]	Norway	National Registry	2082	60	58%	274 Days
Schellenberg et al., 2025 (EPILOC) [5]	Germany	Population-based (PCS)	1154	49	34%	1.5 Years
Águila-Gordo et al., 2021 (Spain) [7]	Spain	Geriatric (Age ≥ 75)	240	83.8	45.80%	352 Days
Horne et al., 2024 (Nutrients) [14]	USA	Prospective Registry	205	64.3	63.70%	2.1 Years

**Table 2:** Detailed Methodological Quality Assessment (Newcastle-Ottawa Scale).

Study (Author, Year)	Selection (Max 4★)	Comparability (Max 2★)	Outcome (Max 3★)	Total Score	Quality Level
N3C Study (Salah, 2022) [9]	★★★★	★★	★★	8 Stars	High
VA Registry (Xie, 2022) [10]	★★★★	★★	★★★	9 Stars	High
Singapore Study (Wee, 2025) [11]	★★★★	★★	★★	8 Stars	High
Ottawa Study (Corrales-Medina, 2025) [6]	★★★	★	★★★	7 Stars	High
Kidney Transplant (Bowring, 2025) [12]	★★★★	★★	★★	8 Stars	High
EPILOC Study (Schellenberg, 2025) [5]	★★★★	★★	★★	8 Stars	High
Norway Registry (Øvrebotten, 2025) [13]	★★★★	★	★★	7 Stars	High
Spain Geriatric (Águila-Gordo, 2021) [7]	★★★	★	★★	6 Stars	Fair
Nutrients Study (Horne, 2024) [14]	★★★	★	★★	6 Stars	Fair

**Table 3:** Individual Criteria Scores for Methodological Quality Assessment.

Study (Author, Year)	S1	S2	S3	S4	C1	O1	O2	O3	Total Score	Quality Level
Salah et al. (2022) [9]	★	★	★	★	★★	★	★	—	8	High
Xie et al. (2022) [10]	★	★	★	★	★★	★	★	★	9	High
Wee et al. (2025) [11]	★	★	★	★	★★	★	★	—	8	High
Corrales-Medina (2025) [6]	★	★	★	—	★	★	★	★	7	High
Bowring et al. (2025) [12]	★	★	★	★	★★	★	★	—	8	High
Schellenberg (2025) [5]	★	★	★	★	★★	★	★	—	8	High
Øvrebotten et al. (2025) [13]	★	★	★	★	★	★	★	—	7	High
Águila-Gordo (2021) [7]	★	★	★	—	★	★	★	—	6	Fair
Horne et al. (2024) [14]	★	★	★	—	★	★	★	—	6	Fair

**Table 4:** Subgroup Analysis and Sources of Heterogeneity.

Subgroup Category	Studies Included	Number of Patients (N)	aHR (95% CI)	Clinical Justification
General Population	N3C, VA, Singapore [11]	N = 470,243	1.36–1.72	Large-scale registries representing the average risk in the broader community.
Immunocompromised	Bowring 2025 (Kidney) [12]	N = 778	2.32 (1.25–4.30)	Represents the highest risk profile due to immune suppression and high comorbidity.
Comparative Risk	Øvrebotten 2025 (Norway) [13]	N = 2082	0.53 (0.36–0.78)	Risk is lower relative to those who recovered from other severe pneumonias
Low-Risk/Healthy	Horne 2024 (Nutrients) [14]	N = 205	0.70 (0.24–2.05)	Registry of patients with specific lifestyle factors showing a non-significant risk.

## Data Availability

The datasets analyzed during the current study are available in the primary research articles cited in the References section. The statistical code used for the meta-analysis in MedCalc v23.4.5 and the standardized data extraction Excel sheet are available from the corresponding author upon reasonable request.

## Supplementary Materials

The following supporting information can be downloaded at: https://www.mdpi.com/article/10.3390/jcm15072665/s1, File S1: Completed PRISMA 2020 Checklist (detailing the location of all required reporting items); File S2: Detailed Search Strategies for PubMed/MEDLINE and Scopus (including full Boolean strings, MeSH terms, and applied filters through 1 January 2026).
