# Supplementary material for: Incident Heart Failure Risk Following COVID-19 Recovery: A Systematic Review and Meta-Analysis
Source: J Clin Med. 2026 Apr 1;15(7):2665. doi: 10.3390/jcm15072665 (PMC13072790; doi:10.3390/jcm15072665)
Supplement: Supplementary file 1 [file jcm-15-02665-s001.zip › Search Strategy.pdf]

This document details the comprehensive search strategy used to identify studies for the systematic review and meta-analysis of heart failure risks in the post-acute phase of COVID-19.

1. Search Overview

- Databases: PubMed/MEDLINE and Scopus.
- Search Period: From the inception of the pandemic to January 2026.
- Language: English.
- Design Filters: Limited to original observational studies (prospective and retrospective cohorts) and clinical trials. Studies were further limited to those with a minimum follow-up period of at least six months post-index infection to satisfy the "long-term" prognostic requirement of the Special Issue.

2. Database-Specific Search Strings

Database 1: PubMed/MEDLINE

Search Date: Through January 2026

("SARS-CoV-2"[MeSH Terms] OR "COVID-19"[MeSH Terms] OR "SARS-CoV-2" OR "COVID-19" OR "Post-Acute Sequelae of COVID-19" OR "Long COVID" OR "PASC") AND ("Heart Failure"[MeSH Terms] OR "Heart Failure" OR "ventricular dysfunction" OR "cardiac dysfunction" OR "congestive heart failure" OR "new-onset heart failure") AND ("Prevalence"[MeSH Terms] OR "Incidence"[MeSH Terms] OR "Hazard Ratio" OR "Prognosis" OR "Long-term" OR "Follow-up" OR "incident")

Applied Filters: Humans; Adult (19+ years); English; Observational Study; Clinical Trial.

Database 2: Scopus

Search Date: Through January 2026.

TITLE-ABS-KEY (("SARS-CoV-2" OR "COVID-19" OR "coronavirus" OR "long-covid") AND ("heart failure" OR "ventricular dysfunction" OR "cardiac outcomes" OR "incident heart failure") AND ("hazard ratio" OR "incidence" OR "prognostic" OR "long-term" OR "follow-up"))

Refinement: Results limited to "Article" and "Review" (reviews were manually screened for primary sources)

3. Manual Search and Citation Mining

To ensure the completeness of this meta-analysis and capture large-scale registry data often referenced in secondary literature, we performed the following:

- Registry Mining: Manual screening of results from the U.S. Veterans Affairs (VA) COVID-19 Registry, the National COVID Cohort Collaborative (N3C), and UK Biobank datasets.
- Backward Citation Tracking: Review of reference lists of high-impact systematic reviews and meta-analyses (e.g., Zuin et al., 2022) to identify primary studies published during the early pandemic.
- Forward Citation Tracking: Use of Google Scholar to identify recent (2024–2026) articles citing established "gold standard" cohorts

4. Study Selection Process (PECO Framework)

In accordance with the PRISMA 2020 guidelines, studies were selected based on the following criteria:

- Population: Adult patients (≥ 18 years) with confirmed SARS-CoV-2 infection history.
- Exposure: Recovery from the acute phase of infection (evaluated >30 days post-index).
- Comparison: Non-COVID-19 control groups (contemporary or historical) or other respiratory viral infection cohorts (Influenza/RSV).
- Outcome: Incident (new-onset) heart failure or cardiac structural changes confirmed via clinical adjudication, ICD-10 codes, or imaging (CMR/Echocardiography)

5. Management of Exclusions

Studies were excluded from the final synthesis if they:

- Included patients with a history of heart failure prior to COVID-19 infection.
- Focused exclusively on acute, in-hospital complications without long-term follow-up.
- Provided no original data (e.g., editorials, mini-reviews, protocols)

6. Data Synthesis and Statistical Analysis

- Analysis Model: A random-effects model was used for pooling hazard ratios (aHR) due to the anticipated high clinical and statistical heterogeneity (I^2 > 75%) across diverse populations, such as general population registries and specialized kidney transplant cohorts.
- Software: Statistical analysis was performed using MedCalc version 23.4.5.
